# *Helicobacter pylori* Genomic Microevolution during Naturally Occurring Transmission between Adults

**DOI:** 10.1371/journal.pone.0082187

**Published:** 2013-12-10

**Authors:** Bodo Linz, Helen M. Windsor, John P. Gajewski, Caylie M. Hake, Daniela I. Drautz, Stephan C. Schuster, Barry J. Marshall

**Affiliations:** 1 Department of Biochemistry and Molecular Biology, Pennsylvania State University, University Park, Pennsylvania, United States of America; 2 Center for Comparative Genomics and Bioinformatics, Pennsylvania State University, University Park, Pennsylvania, United States of America; 3 School of Pathology, University of Western Australia, Crawley, Western Australia, Australia; 4 Singapore Centre on Environmental Life Sciences Engineering, Nanyang Technological University, Singapore, Singapore; University of Hyderabad, India

## Abstract

The human gastric pathogen *Helicobacter pylori* is usually acquired during childhood and, in the absence of treatment, chronic infection persists through most of the host's life. However, the frequency and importance of *H. pylori* transmission between adults is underestimated. Here we sequenced the complete genomes of *H. pylori* strains that were transmitted between spouses and analysed the genomic changes. Similar to *H. pylori* from chronic infection, a significantly high proportion of the determined 31 SNPs and 10 recombinant DNA fragments affected genes of the *hop* family of outer membrane proteins, some of which are known to be adhesins. In addition, changes in a fucosyltransferase gene modified the LPS component of the bacterial cell surface, suggesting strong diversifying selection. In contrast, virulence factor genes were not affected by the genomic changes. We propose a model of the genomic changes that are associated with the transmission and adaptation of *H. pylori* to a new human host.

## Introduction

*Helicobacter pylori* is a human gastric pathogen that colonizes the stomach and duodenum. This bacterium, which is one of the most diverse bacterial species, infects more than half of all humans and is geographically ubiquitous. The association between humans and *H. pylori* is very old because, humans have likely been infected by *H. pylori* since their origin [1]. *H. pylori* accompanied humans during the migrations out of Africa ~60,000 years ago [2], and geographic separation between human populations, and therefore also *H. pylori* populations, have resulted in clusters of bacterial strains that are specific for the large continental areas [1–4]. 

The great genetic diversity in *H. pylori* is caused by an extraordinarily high mutation rate [5,6] that can in part be attributed to the lack of several mutation repair genes that are known from other bacterial species [7]. In addition, infection with multiple strains which occurs occasionally in industrialized countries and occurs commonly in developing countries [8–12] enables recombination between strains, and *H. pylori*'s high recombination rate makes a site three times more likely to be substituted by recombination than by mutation [5]. *H. pylori*'s ability to form aberrant genomic rearrangements and to incorporate non-homologous DNA further enhances the genetic diversity by facilitating gene acquisition and gene loss. All these mechanisms result in a remarkable *H. pylori* diversity, even within a single host [11–13], and a genome in which only ~70% of the, on average, 1560 genes belong to the core genome while the other ~400 to 500 genes per strain are strain specific and variably present [14]. 

Several studies have focused on the genomic changes in *H. pylori* isolates that have been recovered several years apart from patients with chronic infection. For example, whole genome microarray analyses identified events of gene acquisition and gene loss [15,16], and sequencing of gene fragments revealed point mutations (SNPs - Single Nucleotide Polymorphisms) and import of DNA fragments (CNPs - Cluster of Nucleotide Polymorphisms) from co-colonizing *H. pylori* strains [5,17]. Likewise, sequencing of the *cag* pathogenicity island (*cag* PAI) and the *tfs3* type IV secretion systems from consecutive strains revealed SNPs, insertions, deletions and recombinant DNA fragments [18]. On average, the number of gene fragments with genetic changes (mutation or recombination) increased with the sampling interval suggesting that sequence differences accumulated in a clock-like manner, an observation that was also confirmed by whole genome sequences of paired *H. pylori* isolates that were sequentially obtained 3 and 16 years after the initial isolate [6]. The genome analysis of *H. pylori* from chronically infected patients revealed a wide variation of accumulated SNPs and CNPs in the individual strain pairs, as 27 - 232 SNPs and 16 - 441 CNPs were identified. The combined length of CNPs differed between a minimum of about 4 kb to a maximum of 355 kb, possibly depending on the frequency and duration of co-infection with different *H. pylori* strains. Strikingly, genes encoding outer membrane proteins (OMPs) of the *hop* family were at significantly increased frequency among the imported DNA fragments, suggesting diversifying selection [6].

Sequence comparisons of single genes from *H. pylori* isolated from family members revealed identical sequences, point mutations and sequences from unrelated strains [5,11,19,20], while whole genome array studies discovered variable presence of genes that indicated gain and loss of a limited set of genes during intra-familial transmission [20,21]. Most of the variably present genes were coding for proteins with unknown function or for restriction and modification enzymes and only very few outer membrane protein genes [20], unlike genomic changes during chronic infection, a significantly high proportion of which affected OMP genes [6]. However, these differences might be due to the technical limitations of array studies that are usually not sensitive enough to detect point mutations or recombinant DNA fragments. Thus, little is known about the genomic changes that occur during *H. pylori* transmission to a new human host, particularly due to the lack of complete genome sequences. Here we sequenced the complete genomes of two *H. pylori* strains that were isolated from spouses. The genome sequences of these bacterial strains were so similar that they suggested *H. pylori* transmission between the adults, providing the opportunity to analyse the genomic changes that are associated with the adaptation of *H. pylori* to a new human host.

## Results

### *H. pylori* strains from spouses

During a clinical re-infection study that was performed with written informed consent at the Sir Charles Gairdner Hospital, Nedlands, Western Australia, antral gastric biopsy specimens were taken from human volunteers during gastroendoscopy. The results of the re-infection study will be published elsewhere (B.J. Marshall et al, in preparation; B. Linz et al, in preparation). At the time of the final visit of the re-infection experiment, a study participant's spouse had developed symptoms that were similar to those experienced by the volunteer. The clinical symptoms suggested *H. pylori* infection of the spouse, which was confirmed by a positive C^14^ urea breath test (UBT). Subsequently, the spouse underwent gastroendoscopy during which a biopsy specimen was obtained. We cultivated *H. pylori* from the biopsy material obtained from both partners and, after single colony purification, isolated DNA to analyse the bacterial strains. Despite the current consensus that *H. pylori* is predominantly acquired during childhood, we hypothesized that *H. pylori* transmission between the spouses had occurred.

### MLST, RAPD and AFLP analysis

We PCR amplified and sequenced the seven MLST housekeeping genes *atpA*, *efp*, *mutY*, *ppa*, *trpC*, *ureI* and *yphC*. The sequences of all seven gene fragments were identical between study participant's *H. pylori* strain BM012A and the spouses' strain BM012S. In addition, RADP and AFLP analyses (Figure S1) confirmed that the two strains were very similar, because the RAPD patterns obtained from three different primers were almost identical. Likewise, the AFLP analysis revealed only a single additional band in strain BM012S in comparison to BM012A (Figure S1), while unrelated *H. pylori* isolates possessed completely different patterns. Thus, even though the strains were not identical, they were highly related, and one strain likely descended from the other.

### Genome sequencing

**Figure 1:**
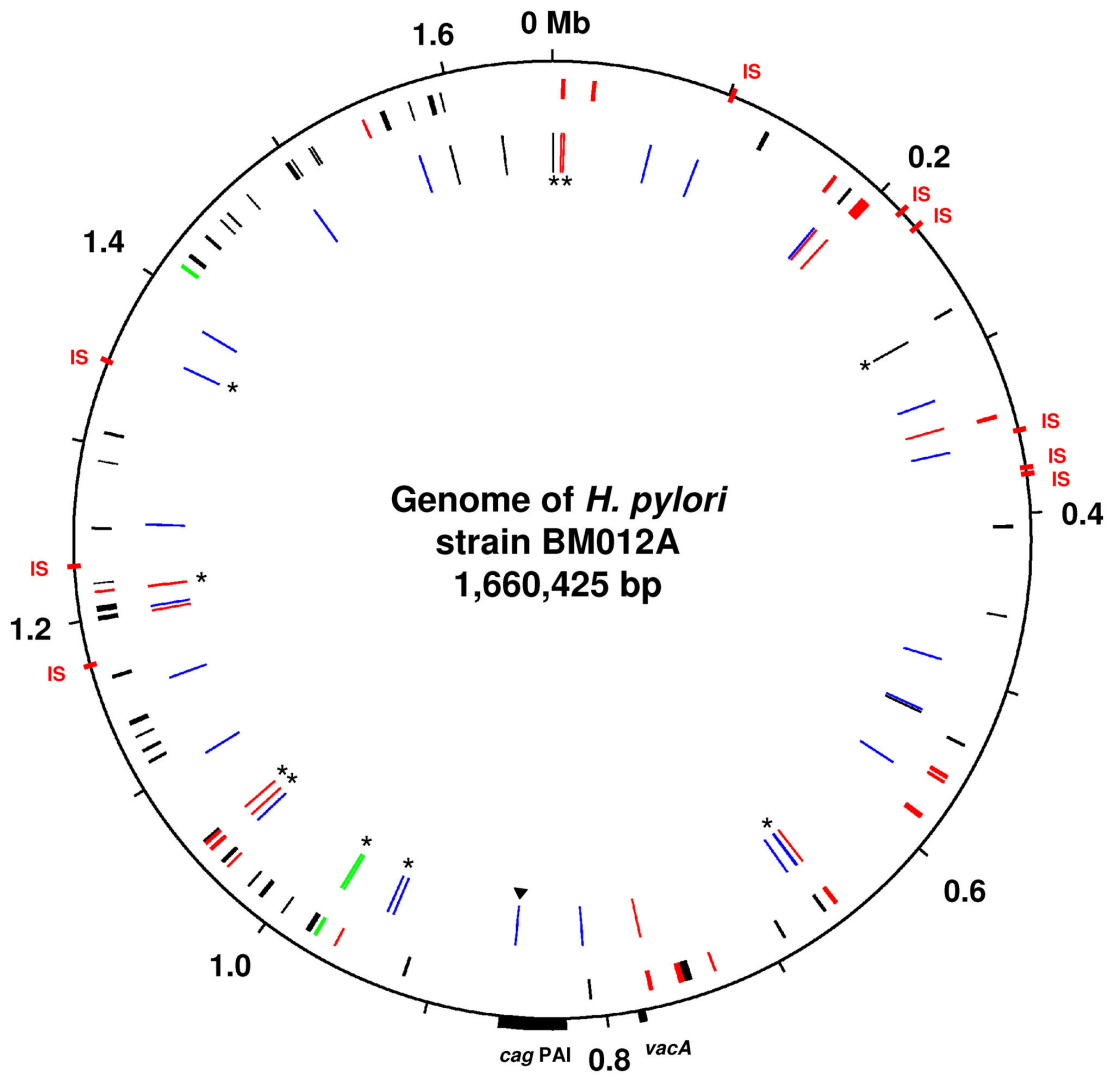
Genomic changes that accumulated in the *H. pylori* genome following transmission between spouses. Circle 1: Genome of strain BM012A with ticks every 0.1 Mb and the positions of the *vacA* gene, the *cag* PAI and IS607 (IS). Circle 2: Distribution of OMP genes of the hop family (red), other OMP genes (black) and fucosyltransferases (green) in the genome of *H. pylori* strain BM012A. Circle 3: Distribution of the 31 SNPs and 10 CNPs (asterisks) that accumulated in the *H. pylori* genome following transmission between spouses. Red - changes in OMP genes; Blue - changes in housekeeping genes; Green - changes in the fucosyltransferase gene *fucT*; Black - changes in non-coding regions; Triangle - the nonsense mutation in the cagD gene.

We determined the complete genome sequences of both *H. pylori* strains using the Roche 454- Titanium FLX sequencer. The genomes were sequenced to 83-fold (BM012A) and 136-fold (BM012S) coverage. The sequences were assembled *de novo*, and the gaps between the 51 contigs of strain BM012A and the 47 contigs of strain BM012S were closed by PCR and Sanger sequencing. Strains BM012A and BM012S possessed highly related genomes with very long stretches of complete sequence identity, which is extremely unlikely to occur between unrelated isolates. The genome of strain BM012A had a length of 1,660,425 bp. Due to a limited number of genomic changes (see below), the genome of strain BM012S was slightly larger (1,660,469 bp). Both genomes possessed the *cag* PAI and a presumably non-functional *vacA* gene that was inactivated by a frameshift. The genomes each contained two copies of the OMP gene *hopS* that encodes the adhesin BabA, and nine copies of the insertion element *IS*607 (Figure 1).

### Genomic changes: Point Mutations

In comparison to the genome of strain BM012A, the genome of strain BM012S possessed 31 single nucleotide polymorphisms (SNPs, separated from the next sequence difference by >200 bp of identical sequence on both sides [6]), confirming that the two strains were very similar (Figure 1, Table 1, Table S1). Three SNPs were located in non-coding regions and 28 SNPs affected genes resulting in 7 synonymous and 21 non-synonymous mutations. 21 SNPs affected housekeeping genes and genes encoding hypothetical proteins while 7 SNPs were located in OMP genes. 21 of the 31 SNPs were substitutions, six were insertions and four were deletions. Two of the ten indels were located in non-coding regions, whereas five truncated the reading frames of the OMP gene *hofH*, of both copies of the OMP gene *hopS*, of the iron regulated OMP gene *frpB*, and of the ferric uptake protein gene *fur*. Two indels enabled gene expression of the OMP gene *hopT* and of the RNA-binding protein gene *jag* by restoring the reading frames, and one merged the reading frames of two small hypothetical protein genes (Table S1). 

**Table 1:** Single nucleotide polymorphisms in the genome of *H. pylori* strain BM012S.

	Gene function (number of SNPs)			
SNPs	Total	Housekeeping	Hypothetical	OMP
Non-coding	3	-	-	-
Synonymous	7	7	-	-
Non-synonymous	21	12	2	7
Total	31	19	2	7

### Genomic changes: Intra-genomic translocation

In addition to the 31 SNPs, the genomes differed by 10 clusters of polymorphic nucleotides (CNPs, two or more sequence differences separated by <200 bp and flanked by >200 bp identical sequence on both sides [6]). Two of the recombinant DNA fragments likely originated by intra-chromosomal translocation of paralogous gene sequences: the OMP gene *hopK* was the source of a 765 bp recombinant fragment in the OMP gene *hopJ*, and 248 bp in the N-terminal region of the fucosyltransferase gene *fucT* were replaced by a fragment from the fucosyltransferase gene *fucU* (Table 2), thereby facilitating the fucosyltransferase expression that was switched OFF in strain BM012A. 

**Table 2:** CNPs in the genome of the derived *H. pylori* strain BM012S.

No.	Genome Position in BM012A	Gene and function	Change	Effect on gene expression
01	164	non-coding	24 to 25 DNA repeats TGATTAG	
02	7288	*hopZ*, OMP HopZ	9 to 10 CT dinucleotide DNA repeats	OFF → ON
03	281036	non-coding	9 to 10 DNA repeats ATACATAA	
04	660180	Poly E-rich protein	5 to 6 DNA repeats TACATCTTGCGGTATTTC	no change, ON
05	940992	Beta-1,4-galactosyltransferase	8 to 9 DNA repeats GATGAACTCACAGGAAAATAC	no change, ON
06	973130 to 973377	*fucT*, Alpha-1,3-fucosyltransferase	248 bp translocation from *fucU* fucosyltransferase	OFF → ON
07	1049893	*hopO*, OMP HopO	7 to 8 CT dinucleotide DNA repeats	ON → OFF
08	1056321 to 1056322	*hopP*, OMP HopP	10 to 9 CT dinucleotide repeats	ON → OFF
09	1215067 to 1215831	*hopJ*, OMP HopJ	765 bp translocation from OMP gene *hopK*	no change, ON
10	1360710	Hypothetical protein	14 to 15 DNA repeats CATTCAAT	correct reading frame uncertain

### Genomic changes: Length polymorphisms in repetitive DNA sequences

The other eight CNPs consisted of length variations in repetitive DNA units, of which seven increased the length of the repeats. The changes included the variation of the CT dinucleotide repeat lengths in the leader peptide regions of the OMP genes *hopP* and *hopO*, switching the expression OFF, and *hopZ*, switching the expression ON. Another change in the coding region of a beta-1,4-galactosyltransferase added a 21-bp DNA repeat unit coding for the peptide DELTGKY, resulting in nine consecutive repeat units in this gene. Two of the length polymorphisms in repetitive DNA sequences were located in non-coding regions. The remaining two changes in repetitive DNA stretches affected a housekeeping gene and a gene whose function is currently unknown (Table 2). Thus, all CNPs likely originated from intra-chromosomal translocation, and none from import of foreign DNA. 

### Diversifying selection for altered bacterial surface structures

Eleven of the 41 genomic changes affected OMP genes, which is significantly more than expected given only 65 OMP genes among the 1591 coding sequences in the genome (Fischer's exact test, *P* = 0.000009). Among the changes, five out of ten CNPs affected bacterial surface structures (*P* = 0.000276), namely switching expression of the OMP gene *hopZ* ON, switching expression of the OMP genes *hopO* and *hopP* OFF, and replacing a stretch of 765 bp of the OMP gene *hopJ* by a translocated fragment of the OMP gene *hopK*. A fifth CNP altered the sequence of the fucosyltransferase gene *fucT*, facilitating fucosylation of the LPS. In addition to CNPs, 7 out of 21 non-synonymous SNPs were located in OMP genes (*P* = 0.000112), but none of the synonymous SNPs (Table 1, Table S1). Among those seven SNPs in OMP genes, two deletions resulted in one frameshift each in the two copies of the *hopS* (*babA*) gene, abolishing expression of this adhesin. The third deletion truncated the OMP gene *hofH*, and an adenine nucleotide deletion next to the CT dinucleotide repeat region in the N-terminus of the *hopT* (*babB*) gene put this gene into the correct reading frame, enabling translation of this OMP. In addition, a nucleotide insertion fragmented the iron regulated OMP gene *frpB*. Taken together, the genomic changes altered the expression of six different OMP genes (*hopS*, *hopO*, *hofH* and frpB→ OFF, *hopT* and hopZ → ON), generated single amino acid changes in two OMPs (HopI and HopZ), and generated a chimerical OMP gene by replacing a 765 bp fragment of the *hopJ* gene (69% of the total gene) by *hopK* sequence (Table 1, Table 2, Table S1). Besides changes in OMPs, translocation of a fucosyltransferase gene fragment enabled modification of the LPS.

## Discussion

### *H. pylori* transmission between spouses

There is general consensus in the literature that *H. pylori* is usually acquired in early childhood, which is supported by the high prevalence of *H. pylori* among children [22–25], and that *H. pylori* infection subsequently endures through most of the host’s life. However, the rates of acquisition and the age at which this occurs vary between developing and developed countries [26], and the frequency and importance of *H. pylori* acquisition in adults might be underrated. Inter-strain recombination that occurs during chronic *H. pylori* infection requires co-colonization with multiple *H. pylori* strains [6,17] that are acquired from other hosts, including other adults. Moreover, previous studies of *H. pylori* strains isolated from couples in Taiwan and in Western Australia estimated that the partners have an up to 50% chance of carrying each other’s strains [13,27], suggesting frequent intra-familial transmission of *H. pylori* between adults. Similarly, the very similar genomes of the *H. pylori* strains BM012A and BM012S that we determined here indicated transmission between the spouses. 

The spouse was likely *H. pylori*-free prior to the transmission event, because after a gastroendoscopy a few years earlier, the patient received antibiotic therapy, followed by a clearly negative UBT (0 dpm). Therefore, we hypothesized that the transmission occurred during the course of the re-infection study from the participant to the spouse. Indeed, the identified genomic changes provided evidence that strain BM012S was derived from BM012A. In strain BM012S, a 765 bp fragment of *hopJ* was replaced by *hopK* sequence and a fragment of the *fucT* gene was replaced by *fucU*. Both recombinant DNA fragments were 100% identical to their donor sequences elsewhere in the bacterial genome, implying intra-chromosomal translocation. In contrast, if strain BM012A had originated from BM012S, replacement of the hybrid *hopJ/hopK* and *fucT/fucU* gene fragments by *hopJ* and *fucT* sequences would have required DNA import from co-infecting *H. pylori*. However, the lack of additional recombinant fragments strongly argues against co-infection with another strain. Moreover, strain BM012A possessed functionally intact *hofH*, *frpB*, *fur* and *hopS* genes, all of which were inactivated by frameshift mutations in strain BM012S, indicating that BM012A was the parental strain. Thus, similar to the host jump of *H. pylori* from humans to large felines that gave rise to *Helicobacter acinonychis* [28], the whole genome analysis revealed the direction of the bacterial transmission.

When did the transmission happen? Barry Marshall had experienced symptoms such as non-acidic vomiting, epigastric distension, malaise, and severe halitosis within one week after his self-infection experiment in the 1980's [29]. Similarly, the volunteers of the re-infection study had developed a variety of symptoms also within two weeks after the re-infection (B.J. Marshall et al., in preparation). While the exact time point of the *H. pylori* transmission between the spouses remains uncertain, we can assume that the spouse had contracted *H. pylori* shortly before the onset of symptoms, near the end of the re-infection study.

### Genomic changes during colonization of a new human host

**Figure 2:**
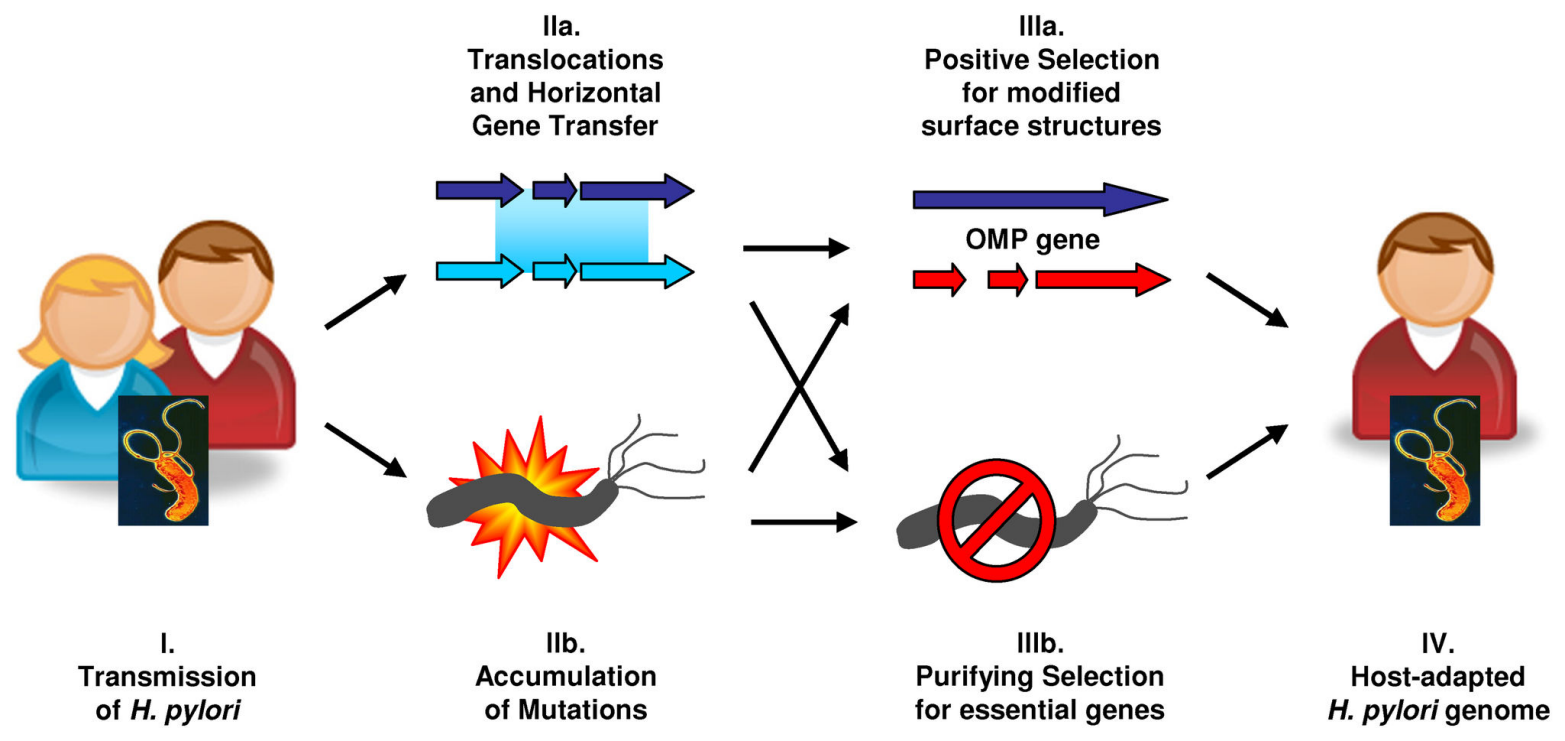
Model of genomic changes associated with *H. pylori* transmission to a new human host. Following transmission (I) of *H. pylori*, mutations (IIb) and recombinant DNA fragments accumulate that result from horizontal gene transfer and intra-chromosomal translocation (IIa). Purifying selection preserves the function of essential genes (IIIb), while diversifying selection by the host's immune system favours growth of bacterial clones with modified surface structures such as OMPs (IIIa). The genomic changes, particularly changes in the expression of bacterial surface components, facilitate adaption of *H. pylori* to a new human host (IV), a dynamic, continuous process that is anticipated to continue throughout the host-pathogen association.

Following *H. pylori* transmission between spouses (Figure 2 I), ten CNPs had accumulated, indicating recombination (Figure 2 IIb). However, none of the fragments seem to have arisen from import of foreign DNA but rather originated by translocation events. Import of DNA from unrelated *H. pylori* depends on (transient) co-infection by another strain. In the case of absence of different *H. pylori* as the source for recombination, uptake and incorporation of DNA still occurs, but it is limited to DNA that was released from dead sister cells. Because the donors and recipients DNAs are basically identical, those recombination events are usually not detectable, with the exception of translocated DNA fragments that have replaced homologous DNA sequences in other regions of the bacterial chromosome or of length polymorphisms in DNA tandem repeats.

Even though recombination was limited to intra-chromosomal translocation, four out of ten CNPs changed the expression of OMP genes, similar to the significantly increased frequency of imports of OMP genes of the *hop* family in *H. pylori* during chronic infection [6]. In addition, the DNA import into the *fucT* gene (Table 2) switched the *fucT* expression ON, thereby facilitating posttranslational fucosylation of the LPS O-antigen to generate Lewis antigens, structures also found on human epithelial cells. A further change of the *H. pylori* antigenic properties possibly resulted from an increase of the C-terminal heptad repeat region of a beta-1,4-galactosyltransferase gene from eight to nine repeat units (Table 2). The variable number of DELTGKY tandem repeats might directly affect the length of the N-acetyl-lactosamine polysaccharide O-chain backbone of the LPS [30], similar to the *H. pylori* alpha-1,3-fucosyltransferases in which the lengths of the DDLRVNY tandem repeats in the homo- and heterodimers of the enzyme determine the number of N-acetyl-beta-lactosamine units that get glycosylated in the O-antigen chain [31]. In addition to CNPs, 31 SNPs accumulated in the genome of strain BM012S (Figure 2 IIa), seven of which (~23%) changed the protein sequence and/or expression of OMPs. Thus, numerous changes in OMPs and carbohydrate compounds of the bacterial cell envelope suggest selection against immunogenic surface structures (Figure 2 IIIb).

Surprisingly, no changes were found in *H. pylori* virulence factors, despite previous studies that described microevolution in essential *cag* PAI genes such as *cagA*, *cagE* or *cagY* and in the vacuolating cytotoxin gene *vacA* in successive isolates that were obtained three, ten or sixteen years apart from patients with chronic infection [6,18,32]. We note that *vacA*, as well as the ulcer-associated restriction endonuclease gene *iceA*, were already pseudogenized in the parental strain and were therefore likely not under selection by the host's immune system. Furthermore, the duodenal ulcer-promoting gene *dupA* was missing from the genomes. In contrast, the *cag* PAI sequence suggested the presence of a functional, and therefore potentially immunogenic, type IV secretion system. Yet, the only observed change in the *cag* PAI of strain BM012S was a substitution that truncated the *cagD* gene by the introduction of a nonsense mutation (Figure 1, Table S1). However, to our knowledge, the CagD protein is not essential for the function of the *cag* PAI type IV secretion system, as a *cagD* knockout mutant only marginally affected the translocation of CagA and the induction and secretion of interleukin-8 in AGS cells [33]. 

In contrast to OMP genes, housekeeping genes contained both synonymous and non-synonymous mutations. No core genes were affected by frameshift mutations, presumably because of purifying selection against deleterious mutations in essential genes. Moreover, some disadvantageous substitutions also seemed to be removed by purifying selection (Figure 2 IIIa) because only 10 out of the 17 substitutions (59%) in housekeeping genes were non-synonymous (Table S1), slightly less than the theoretical 75% of all possible substitutions. 

We note the remote possibility that some of the observed genomic changes might have originated in *H. pylori* clones in the original human host prior to the transmission event, reflecting the *H. pylori* microdiversity in and continuous adaptation to a single host. However, we suppose that the vast majority of the changes are associated with the transmission and adaptation to a new host, because all SNPs and CNPs were different from those that were identified in the output strains from the re-infection experiment (B. Linz et al., in preparation).

Taken together, following transmission of *H. pylori* between spouses (Figure 2 I), we observed accumulation of mutations (Figure 2 IIb) that affected housekeeping genes as well as genes encoding surface structures of the bacterial cell. We detected recombinant DNA fragments (Figure 2 IIa), that had translocated from elsewhere in the genome, but no imported DNA from other *H. pylori*. We found strong evidence for diversifying selection for modified surface components, including outer membrane proteins (Figure 2 IIIa), as well as signs of purifying selection in housekeeping genes (Figure 2 IIIb). The genomic changes, particularly the changes in the expression of bacterial surface structures, are anticipated to facilitate adaption of the bacterium to the new human host (Figure 2 IV), a dynamic, continuous process that will likely continue throughout the host-pathogen association.

### Genomic changes in comparison to a model of a jump between host species

The genomic changes that occur during *H. pylori* transmission between humans are very similar to those that were thought to be crucial for the successful jump of a bacterial pathogen to a new host species. According to this model, that was based on the whole genome analysis of an *H. pylori* host jump from humans to large felines that resulted in *Helicobacter acinonychis* [28,34], bacterial infection of the epithelium induces a mutation burst which generates many different subpopulations of clonally related bacteria, each with a specific combination of mutated and unaffected genes. Purifying selection removes bacteria with disadvantageous mutations in essential genes from the population, but variants with altered or functionally inactivated genes that encode surface epitopes that are recognized by the host’s immune system, will be selected for. Some of the fragmented genes will subsequently be deleted from the genome given time. Horizontal gene transfer from co-colonizing bacteria might also play a role during the adaptation process, particularly importation of genes that encode surface components of bacterial species that are already adapted to the host. All these changes finally result in a genome that is adapted to the new host [34].

## Materials and Methods

### Bacterial strains and ethics statement

The clinical part of this study was performed with written informed consent at the Sir Charles Gairdner Hospital, Nedlands, Western Australia, under ethics approval 2007-045 by the Human Ethics Committee of this hospital. Antral gastric biopsy specimens were collected during gastroendoscopy from a participant of a re-infection study who has had a presumably lifelong, asymptomatic *H. pylori* infection. At the end of the re-infection experiment that involved two cycles of antibiotic therapy and subsequent re-infection with the volunteer's own *H. pylori* strain, the study participant's healthy spouse had developed symptoms that suggested an infection with *H. pylori*. *H. pylori* infection was confirmed by a positive UBT and a subsequent gastroendoscopy during which a biopsy specimen was taken. The biopsies from the study participant and her spouse were cultivated on Pylori agar plates (Biomerieux, Mercy - l’Etoile, France) from which the *H. pylori* strains BM012A (study participant) and BM012S (spouse) were obtained. After single colony purification, DNA was isolated for whole genome sequencing using the Qiagen Blood and Tissue Kit. The bacterial DNA was sent to the Pennsylvania State University for whole genome sequencing. Since no patient information was provided, the study was approved by the ethics board of the Pennsylvania State University as research not involving human participants (IRB#35170). 

### Strain typing

RAPD (Random Amplified Polymorphic DNA) fingerprinting of *H. pylori* genomic DNA [35] was used to assess the relatedness of the strains by using primers 1247 (5'- AAGAGCCCGT), 1254 (5´-CCGCAGCCAA) and 1281 (5´-AACGCGCAAC) in separate reactions. PCR was carried out in 25µl volumes containing 20ng of genomic DNA, 3mM MgCl_2_, 250µM of each dNTP, 20pmol of primer and 1U of Taq DNA polymerase (Roche Diagnostics, Mannheim, Germany) in the buffer provided. The program consisted of 45 cycles of denaturation at 94°C for 1 min, annealing at 36°C for 1min and extension at 72°C for 1 min, followed by 10 min final extension at 72°C. The products were separated by electrophoresis in a 2% (w/v) agarose gel at 75 V for 2.5h. 

AFLP (Amplified-Fragment Length Polymorphism) fingerprints of *H. pylori* strains were generated by *Hin*dIII digestion of genomic DNA followed by ligation with adapter oligonucleotides as described by Gibson et al. [36]. Briefly, an aliquot containing 5µg DNA was digested overnight (16h) at 37°C with 12 U *Hin*dIII (Roche Diagnostics, Mannheim, Germany) in the manufacturer’s buffer and 5mM spermidine trihydrochloride (Sigma-Aldrich, St. Louis, MO, USA). A 10 µl aliquot of digested DNA was used in a ligation reaction containing a final volume of 23 µl with 0.2µg of each of the adapter oligonucleotides ADH1 (5´-ACGGTATGCGACAG) and ADH2 (5´-AGCTCTGTCGCATACCGTGAG), 1 U of T4 DNA ligase (Roche) and ligase buffer at 37°C for 4 h. Following heat inactivation of the T4 enzyme activity at 80°C for 10 min, the ligated DNA was used as template DNA for PCR amplification in 50µl reactions containing 5 µl of template DNA, 0.2 mM of each dNTP, 0.15µg of H1A primer (5´-GGTATGCGACAGAGCTTA), 1 U of Taq DNA polymerase (Roche Diagnostics, Mannheim, Germany) and 2.5 mM of MgCl_2_ in standard PCR buffer. The amplification consisted of 2 min at 94°C and then 33 cycles of 30sec at 94°C, 30sec at 60°C and 60sec at 72°C. The products were separated by electrophoresis in a 2% (w/v) agarose gel at 75 V for 2.5h. 

PCR amplification and sequencing of the MLST housekeeping genes *atpA, efp, mutY, ppa, trpC, ureI* and *yphC* were performed as previously described, using the primer pairs O_1964 + O_1965 (*atpA*), O_2046 + O_2045 (*efp*), O_1979 + O_1980 (*mutY*), O_3238 + O_3241 (*ppa*), O_1370 + O_1372 (*trpC*), O_3242 + O_3151 (*ureI*), and O_1960 + O_1963 (*yphC*) [2].

### Genome sequencing and assembly

The genomes strains BM012A and BM012S were sequenced to 83-fold (BM012A) and 136-fold (BM012S) coverage on a 454-Roche Titanium FLX sequencer. The sequence reads were assembled *de novo* in Newbler [37], and the genomic gaps in strains BM012A and BM012S were closed by PCR and Sanger sequencing. The genomes were annotated using the RAST Server [38], followed by manual curation. The genome sequences are available in Genbank under the accession numbers CP006888 (BM012A) and CP006889 (BM012S), the sequencing reads are available in the NIH Sequence Read Archive under the accession numbers SRS493238 (BM012A) and SRS493239 (BM012S).

### Genome analysis

The identified nucleotide changes in the strain pair BM012A and BM012S were validated by aligning the individual sequence reads of strains BM012A and BM012S against the reference genome sequences of both strains using gsMapper implemented in Newbler [37]. SNPs (separated from the next sequence difference by >200 bp of identical sequence on both sides) were assumed to be introduced by mutation while clusters of 2 or more polymorphisms (CNPs, separated by <200 bp and flanked by >200 bp of identical sequence on both sides) were likely the result of recombination [6]. The genomic differences (Figure 1) were plotted using GenomeViz [39]. DNA fragments that were presumably introduced by recombination (Table 2) were searched for possible intra-chromosomal DNA donor sources. All recombinant DNA fragments likely arose by intra-chromosomal recombination rather than by DNA import from an unrelated strain.

## Supporting Information

Figure S1 **Similar RAPD and AFPL patterns suggest *H. pylori* transmission between spouses.** (A-C) Similar RAPD patterns of *H. pylori* strains BM012A and BM012S. (B) *Hin*dIII.
AFLP fingerprints of strains BM012A and BM012S reveal a single additional band in strain .
BM012S. lanes: 1 - 100bp ladder; 2 and 3 - strain BM012A; 4 and 5 - strain BM012S.
(PDF)

Table S1 **31 SNPs that accumulated in the genome of strain BM012S.**
(PDF)
