# Supplementary material for: Helicobacter pylori Genomic Microevolution during Naturally Occurring Transmission between Adults
Source: PLoS One. 2013 Dec 10;8(12):e82187. doi: 10.1371/journal.pone.0082187 (PMC3858298; doi:10.1371/journal.pone.0082187)
Supplement: Figure S1 — Similar RAPD and AFPL patterns suggest H. pylori transmission between spouses. (A-C) Similar RAPD patterns of H. pylori strains BM012A and BM012S. (B) HindIII. AFLP fingerprints of strains BM012A and BM012S reveal a single additional band in strain . BM012S. lanes: 1 - 100bp ladder; 2 and 3 - strain BM012A; 4 and 5 - strain BM012S. (PDF) [file pone.0082187.s001.pdf]

Figure S1

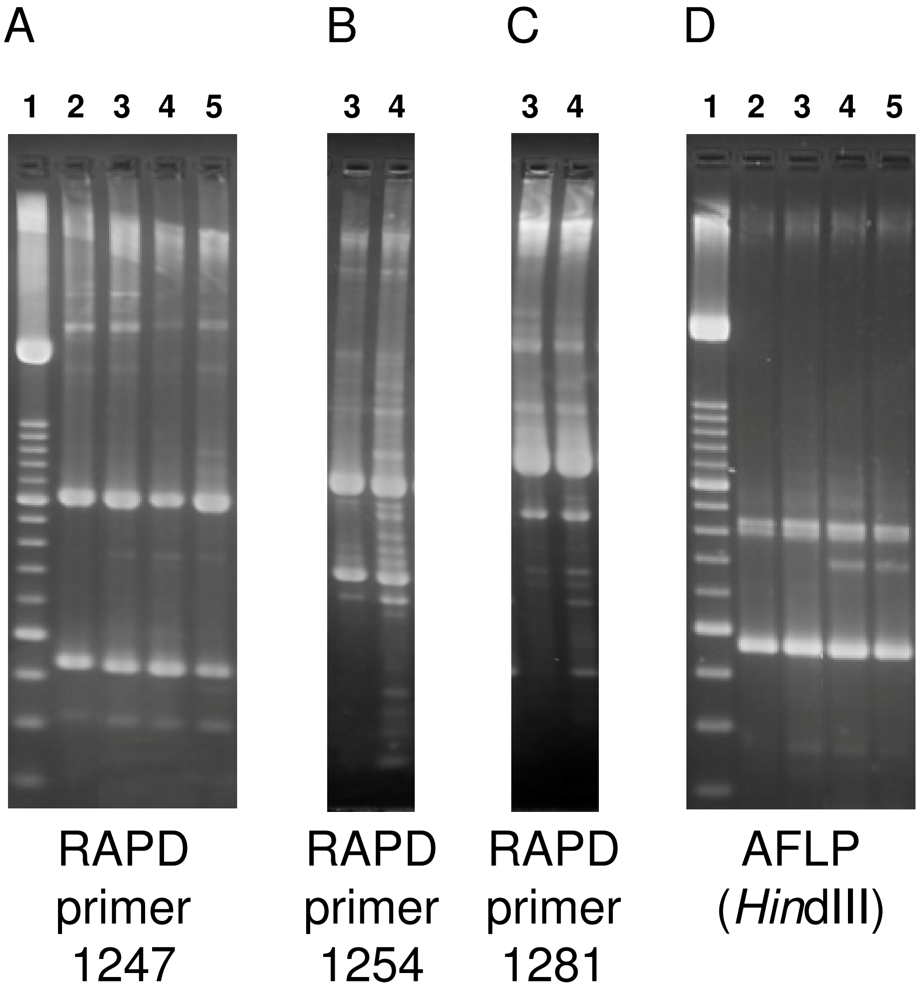

Figure S1. Similar RAPD and AFPL patterns suggest *H. pylori* transmission between spouses. (A-C) Similar RAPD patterns of *H. pylori* strains BM012A and BM012S. (B) *Hin*dIII AFLP fingerprints of strains BM012A and BM012S reveal a single additional band in strain BM012S. lanes: 1 - 100bp ladder; 2 and 3 - strain BM012A; 4 and 5 - strain BM012S.
