# Supplementary material for: Helicobacter pylori Genomic Microevolution during Naturally Occurring Transmission between Adults
Source: PLoS One. 2013 Dec 10;8(12):e82187. doi: 10.1371/journal.pone.0082187 (PMC3858298; doi:10.1371/journal.pone.0082187)
Supplement: Table S1 — 31 SNPs that accumulated in the genome of strain BM012S. (PDF) [file pone.0082187.s002.pdf]

Table S1. 31 SNPs that accumulated in the genome of strain BM012S.						
SNP No.	Genome position in strain BM012A	Nucleotide in strain BM		Amino acid change	Gene	Annotation/Comment
		012A	012A			
SNP01	6919	C	A	non-syn	*hopZ*	OMP HopZ
SNP02	64602	G	A	syn	*dnaB*	Replicative DNA helicase
SNP03	96594	T	G	non-syn		Hypothetical protein
SNP04	184511	C	T	syn	*cspA*	Carbon starvation protein A
SNP05	185758	A	-	non-syn	*hofH*	Frameshift, truncating OMP HofH
SNP06	196072	C	T	non-syn	*hopI*	OMP HopI
SNP07	323044	T	C	syn	*nuoG*	NADH-ubiquinone oxidoreductase chain G
SNP08	342487	A	-	non-syn	*hopT*	OMP HopT (BabB), deletion next to CT dinucleotide repeats switching expression ON
SNP09	358101	G	A	non-syn	*traG*	Conjugal transfer protein TraG
SNP10	493810	C	A	non-syn	*ompR*	Response regulator OMPR
SNP11	530245	C	T	syn	*fabH*	3-oxoacyl-[acyl-carrier-protein] synthase
SNP12	530632	-	T	non-coding		
SNP13	567936	A	G	non-syn	*hcpE*	Beta-lactamase HcpE
SNP14	655338	C	-	non-syn	*hopS*	Frameshift, truncating OMP HopS (BabA)
SNP15	667551	T	C	syn	*ilvC*	Ketol-acid reductoisomerase
SNP16	772592	G	-	non-syn	*hopS*	Frameshift, truncating OMP HopS (BabA)
SNP17	810488	G	A	non-syn	*pgbA*	Plasminogen-binding protein PgbA
SNP18	854282	G	A	non-syn	*cagD*	Nonsense mutation, truncating *cag* PAI protein CagD (non-essential)
SNP19	936267	C	T	non-syn	*cheV*	Chemotaxis protein CheV
SNP20	972864	A	G	non-syn	*fucT*	Alpha (1,3)-fucosyltransferase
SNP21	1044457	G	A	syn	*rpoN*	RNA polymerase sigma-54 factor RpoN
SNP22	1099774	T	A	non-syn	*amiA*	N-acetylmuramoyl-L-alanine amidase
SNP23	1154030	T	C	non-syn		Beta-1,4-galactosyltransferase
SNP24	1199210	-	G	non-syn	*frpB*	Frameshift, truncating iron-regulated OMP FrpB
SNP25	1202219	-	A	non-syn		Frameshift, fusing two hypothetical protein genes
SNP26	1255390	-	A	non-syn	*fur*	Frameshift, truncating ferric uptake regulation protein FUR
SNP27	1387116	G	A	syn	*pheS*	Phenylalanyl-tRNA synthetase alpha chain
SNP28	1495189	-	C	non-syn	*jag*	Insertion restoring the reading frame in RNA-binding protein Jag
SNP29	1572331	G	A	non-syn		Type III restriction-modification system methylation subunit
SNP30	1593255	-	T	non-coding		
SNP31	1627753	G	A	non-coding		
OMP genes are highlighted in red						
